# A Biomechanical Research of Growth Control of Spine by Shape Memory Alloy Staples

**DOI:** 10.1155/2013/384894

**Published:** 2013-11-17

**Authors:** Wei Zhang, Yonggang Zhang, Guoquan Zheng, Ruyi Zhang, Yan Wang

**Affiliations:** Department of Orthopedics, General Hospital of Chinese PLA, Beijing 100853, China

## Abstract

Shape memory alloy (SMA) staples in nickel titanium with shape memory effect are effective for spinal growth control. This study was designed to evaluate the biomechanical properties of the staples and observe the stability of the fixed segments spine after the staples were implanted. According to the vertical distance of the vertebrae, SMA staples of 5, 6.5, and 8 mm were designed. The recovery stress of 24 SMA staples in three groups was measured. The pullout strength of SMA staples and stainless steel staples in each functional spinal unit was measured. Each of the six fresh specimens was divided into three conditions: normal, single staple, and double staples. Under each condition, the angle and torque of spinal movements in six directions were tested. Results show that the differences in recovery stress and maximum pullout strength between groups were statistically significant. In left and right bending, flextion, and extention, the stability of spine was decreased in conditions of single staple and double staples. Biomechanical function of SMA staples was superior to stainless steel staple. SMA staples have the function of hemiepiphyseal compression and kyphosis and scoliosis model of thoracic vertebrae in goat could be successfully created by the fusionless technique.

## 1. Introduction

Idiopathic scoliosis deformity is one of the common diseases in children and adolescents [1, 2]. Its main feature is the three-dimensional deformities of spinal structures before skeletal maturity, including abnormal curvature of the sagittal and coronal surface of spine and axial rotation of vertebrae [3].

Currently, surgical or nonsurgical treatments for the near future or forward to change the natural development course of the disease of scoliosis deformity is used for the treatment of children and adolescents with idiopathic scoliosis deformity with full consideration to the premise of the disease natural history [4, 5]. Bracing and surgical treatments were commonly used dealing with spinal deformity [6, 7]. However, there are still some severe problems and complications because of controlling or correcting deformity in the use of external strength [8, 9]. From the point of physiology, bracing is rational, which could preserve the structure and function of spine [10, 11]. But, for the reason of indirect force, the therapeutic efficacy was not ideal in many cases, and some cases are not suitable to be treated with bracing. Surgical treatment has achieved great advancements in correcting deformity, but it is not a choice for patients; when getting beautiful shapes, they have been deprived of partial functions and had rigid spines [12, 13].

During the growth of spine, multiple organic centers are formed. It is possible to control certain organic centers in modulating spinal growth. At the same time, spinal deformity is to be corrected and partial functions are to be retained [14]. With more investigations of spinal deformity and further developments in minimally invasive surgery of spine, it may be available to control or correct spinal deformity through spinal growth control [15, 16]. 

Hemiepiphyseal compression staples were intrasegmental fixation instrument designed by hemiepiphyseal block technique [17, 18]. In order to deal with the bone bending caused by the imbanlance growth of growth-plates, one side of the epiphyseal plate which grows relatively fast is temporarily stalled so that the other side could grow adequately to correct the skeletal deformities. The hemiepiphyseal block technique could avoid osteotomy, prevent postoperative gypsum fixation and reduce the bed rest [19]. 

Shape memory alloy (SMA) staples were designed according to the measurements of goat's vertebral. Maximum pullout force of the staples with different materials and design, biomechanical properties, stability of the fixed segment, and the impact on the growth of the goat's spine were evaluated in our research. It provided a reference for clinical application.

## 2. Materials and Methods

### 2.1. Design of Shape Memory Alloy (SMA) Staples

 Goat's thoracic has 13 segments, shaped like a human vertebral body, but the middle sag is more obviously. Six female normal goat's (2 to 3 months of age, weight 7–9 Kg) thoracic spine specimens were excluded paravertebral fat, muscle, and other soft tissue retaining ligaments and joint capsule that did not damage the bone structure. Digital X-ray and CT were taken to expel tumors and congenital malformations.

**Figure 1:**
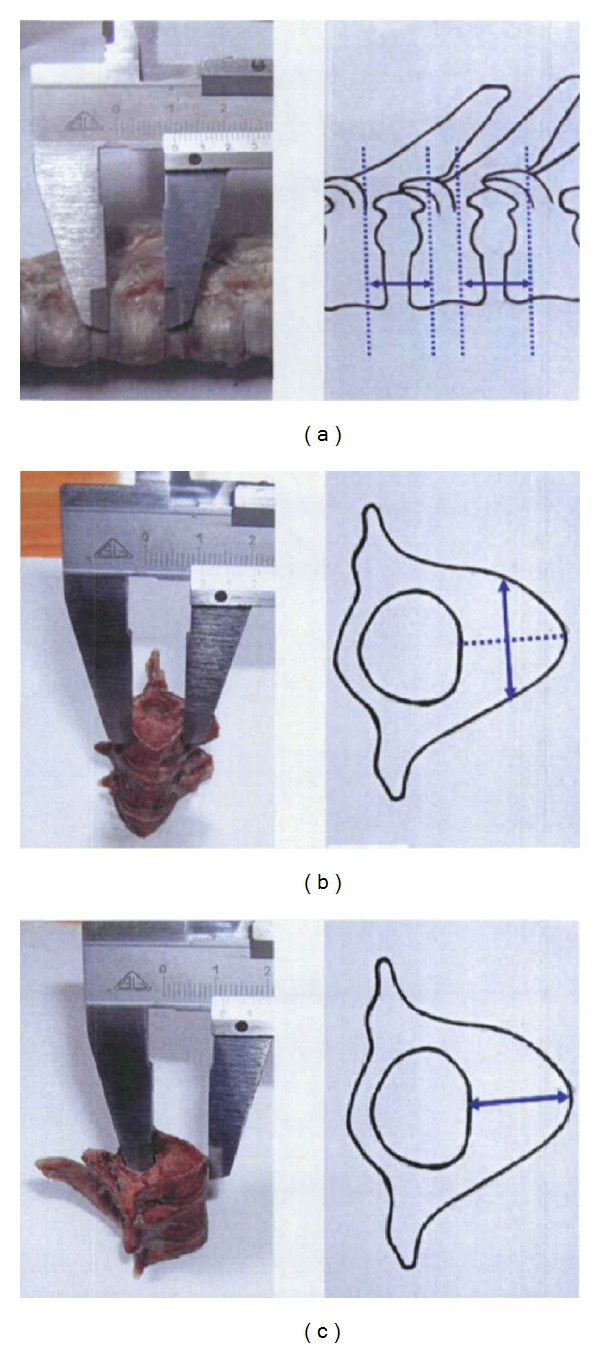
The measurement of the segments in T6–T11. (a) The vertebral body side 1/3 cross-sectional distance of each adjacent segments. (b) The maximum diameter, medial diameter of the 1/3 cross-sectional of T6–T11 vertebrae. (c) The maximum sagittal diameter of the 1/3 cross-sectional of T6–T11 vertebrae.

The vertebral body side 1/3 cross-sectional distance of each adjacent segments in T6–T11 was measured as Figure 1(a). The maximum diameter, medial diameter, and sagittal diameter of the 1/3 cross-sectional of T6–T11 vertebrae were measured as in Figures 1(b) and 1(c). The original shape of orthopedic staples were “C” type which across the intervertebral implant in the 1/3 position of each adjacent vertebral body. Its length is equal to 1/3 cross-sectional distance of each adjacent vertebral body, its depth is between the maximum diameter and medial diameter of the 1/3 cross-sectional of T6–T11 vertebrae, and its width does not exceed the maximum sagittal diameter of the 1/3 cross-sectional of T6–T11 vertebrae.

**Figure 2:**
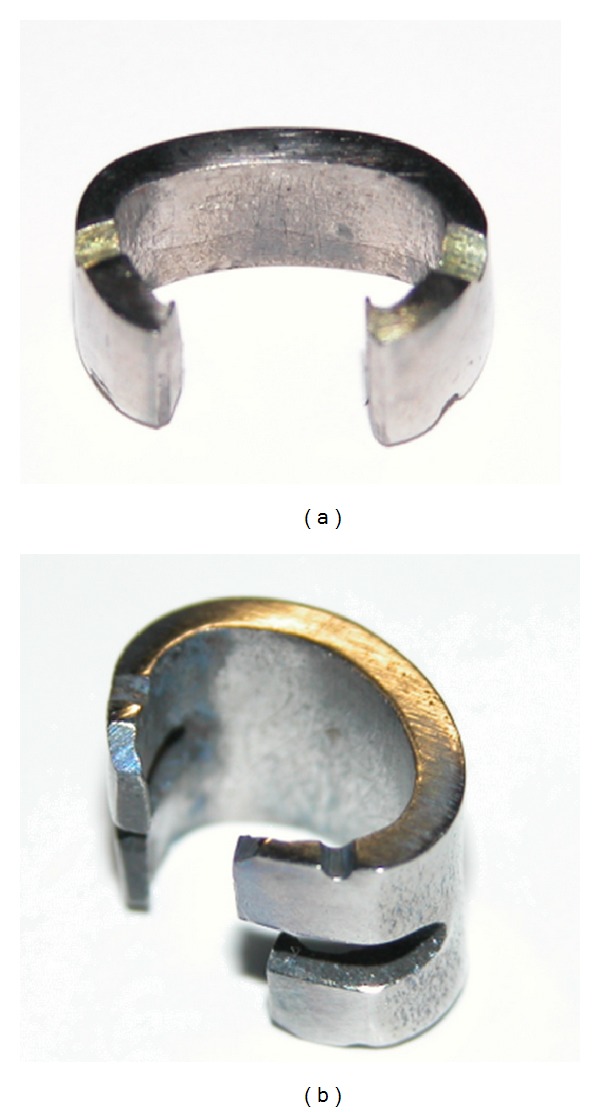
Two kinds of orthopedic staple. (a) Orthopedic staple with two tines. (b) Orthopedic staple with four tines.

According to the above measurement, we designed two kinds of orthopedic staples with two tines or four tines, respectively. Its length was 10 mm, the tine depth was 4 mm and thickness was 2 mm, the width was 2 mm or 4 mm, respectively. Each kind of staples was designed into 3 types which had different distances between the tines as 5 mm, 6.5 mm and 8 mm. In order to increase its antipullout and the antirotation performance, we design groove in the tine of the staples (Figure 2). In accordance with the above dimensions, stainless steel hemiepiphyseal orthopedic staples with two-tine were designed. Its interdental distance is 10 mm. 

### 2.2. Maximum Recovery Stress Test of the Orthopedic Staples

**Figure 3:**
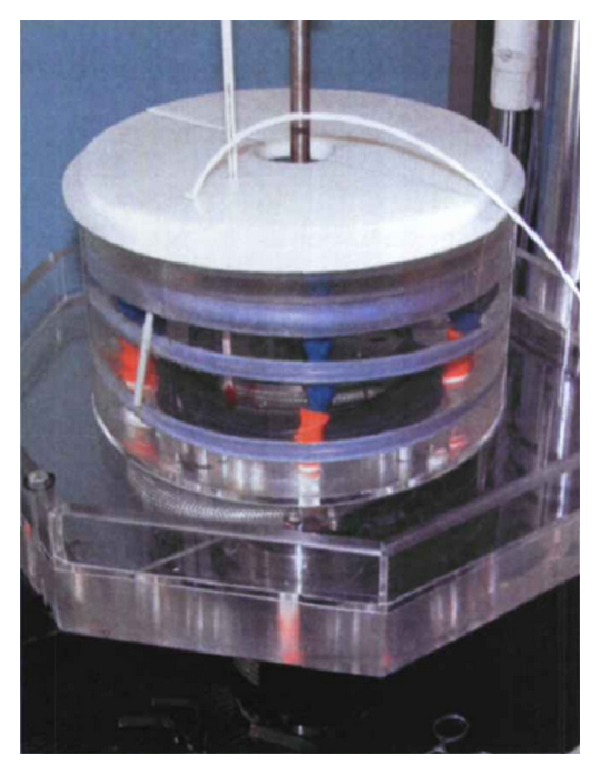
Thermostat water bath.

**Figure 4:**
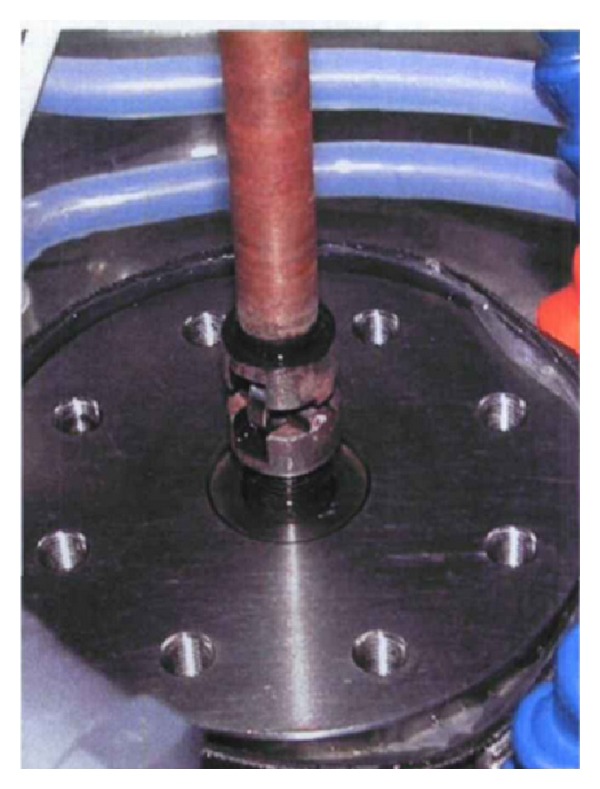
Staples roachback was parallel to the axis of fixture.

 One important indicator for evaluation of shape memory effect of memory alloy is the recovery stress, which is generated by heating to a temperature above the body temperature to restore the original shape of the alloy. This maximum recovery stress test was carried out to evaluate the shape memory effect of the new designed staples. In this study two-tine SMA staples were chosen and divided into three groups: 5 mm group, 6.5 mm group, and 8 mm group. Each group had eight staples. At first, the interdental distance between the tines of orthopedic staples was stretched to 10 mm (measured by vernier caliper) in the ice water mixture; then the staples were placed into the corresponding fixture in thermostat water bath (Figure 3). At last, the two tines were separately clicked into the upper and lower fixture groove and keep the long axis of orthopedic staples parallel to the axis of fixture (Figure 4) to ensure the recovery stresses uniform. The experiments were performed on the MTS858 Mini biomechanical test machine (MTS corp. US), and both ends of the fixture were connected to a computer by the sensor. Before the experiment, the original displacement and the raw data of the recovery stress was reset to zero. Then 55°C hot water was added to thermostat water bath, fully immersing orthopedic staples, and the bath was capped at once to keep the water temperature whose changes were monitored by electronic thermometers.

### 2.3. Test on the Maximum Pullout Strength of SMA Staples

#### 2.3.1. Instruments

Specially designed two-tine stainless steel staples, three kinds of two-tine shape memory alloy hemiepiphyseal compression staples and implantation related instruments: holding pliers, distraction pliers, and vertebral opening device, supplied by Beijing Ming Yuen Laboratory Furniture Co. Ltd. and Beijing HengShi ZiZheng Biotechnology Co. Ltd.

#### 2.3.2. Preparation of the Specimens

Six fresh specimens of the vertebrae (T6–T11) in goats (age 2-3 months) were selected and excluded paravertebral fat, muscle, and other soft tissue retaining ligaments and joint capsule without any impairment on the bone structure. Each specimen of the vertebrae (T6–T11) was cut into three functional spinal units (FSU): T6-T7, T8-T9, and T10-T11, including the adjacent vertebrae and their intervertebral discs, facet joints, and ligaments connecting structure. Each FSU was randomly assigned and recombined into six vertebral body specimens, and every specimen contained the 3 FSUs of T6-T7, T8-T9, and T10-T11. Specimens were saved in −80°C and natural thawed 12 hours before testing [20].

#### 2.3.3. Experiment Grouping

Stainless steel and shape memory alloy hemiepiphyseal compression staples were successively implanted into FSUs, which were divided into four groups: 5 mm group, 6.5 mm group, 8 mm group, and stainless steel staples group, and eight SMA and twenty-four stainless steel staples were tested in each group.

#### 2.3.4. Experiment Process

**Figure 5:**
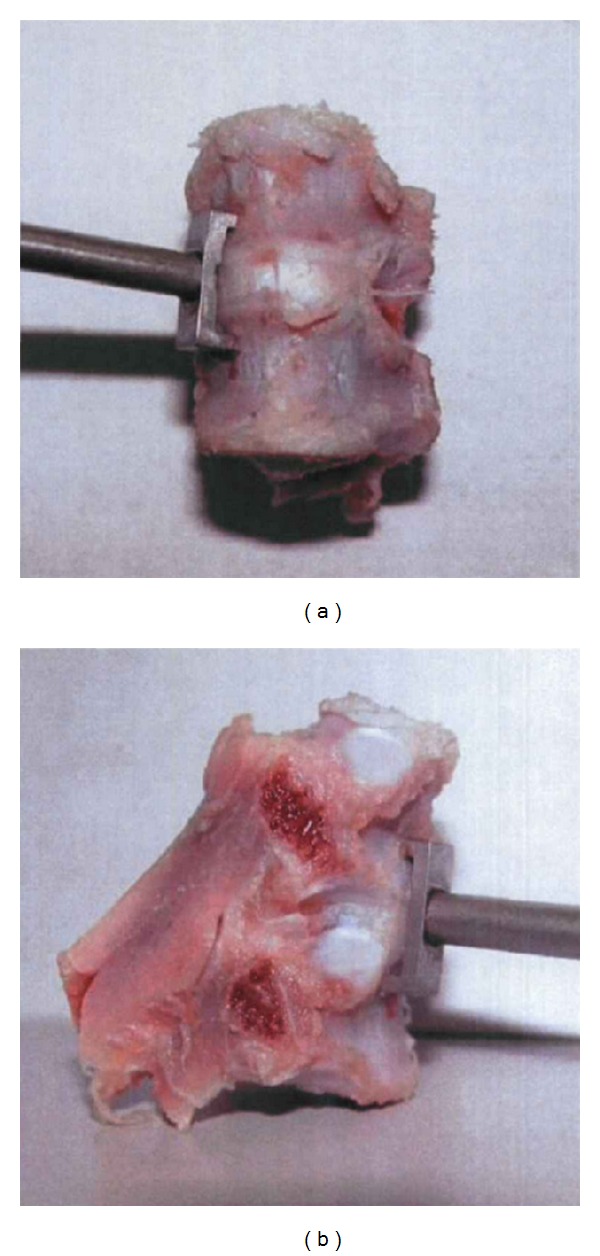
The location from where stainless steel orthopedic staples were implanted in each adjacent vertebrae of FSU across the intervertebral space. (a) The anterior; (b) the lateral.

**Figure 6:**
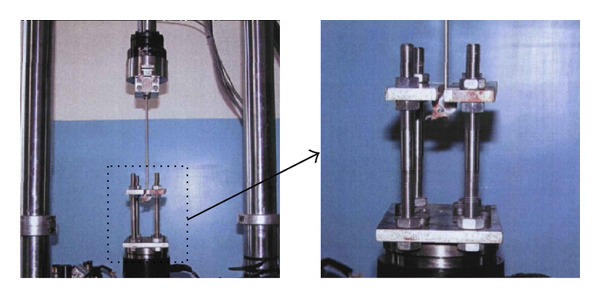
One end of FSU which implanted the stainless steel was fixed in the fixture, while the other end was placed under the steel plate on both sides of the fixture, keeping the long axis of orthopedic staples parallel to the axis of fixture.

*(1) Pull-Out Test of Stainless Steel Hemiepiphyseal Compression Orthopedic Staples.* Twenty-four stainless steel orthopedic staples were randomly assigned and then were implanted in the left and right side of each adjacent vertebrae of FSU from the anterior lateral across the intervertebral space, placed in front of the vertebral transverse process costal fovea. Simultaneously, the transverse process and part of the spinous process of the vertebral body were removed (Figure 5). The experiments were performed on the MTS858 Mini biomechanical test machine, with a 662.20D-03 sensor. After stainless steel orthopedic staples implanted in the FSU, the round rod end of the orthopedic staples was fixed in the fixture, while the FSU was placed under the steel plate on both sides of the fixture, keeping the long axis of orthopedic staples parallel to the axis of fixture. The height and direction of the plate was adjusted to make the steel plate accommodate with the shape of the side structure of the FSU, so that the FSU under the steel plate did not occur lateral sway to make sure that the pullout force was uniform (Figure 6). On the basis of the preexperimental results we set up an experiment machine pull-out speed 1 mm/min, with no limits for maximum tension and the maximal displacement. Before the test, the force between the fixture and the FSU was adjusted through computer software, the original pullout strength and the displacement was reset to zero. The test was stopped as the orthopedic staples were separated from spine unit after loading the pullout force.

*(2) Pull-Out Test of SMA Staples.* In order to compared the pull-out strength of stainless steel and SMA staples under the same conditions, stainless steel orthopedic staples were removed from the vertebral body specimens, and then two SMA staples of 5 mm, 6.5 mm, and 8 mm, respectively, implanted cross into each group of FSU. The center of memory alloy orthopedic staples roachback was clamped by holding pliers and then implanted in the left and right side of each adjacent vertebrae of FSU from the anterolateral across the intervertebral space, the same implantation position with stainless steel staples. And then SMA staples were treated with 55°C hot water to recover their deformation and fix firmly into the vertebral cancellous bone. The following test process was the same as above.

### 2.4. Test on the Stability of SMA Staples

#### 2.4.1. Preparation of the Specimens

Twelve specimens of the vertebrae (T5–T12) obtained from goats of the same age were selected and treated just as described in Section 3.2. Both ends of T5, T12 vertebral body were embedded by polymethyl methacrylate methyl, with upper and lower ends embedded into a cylindrical to ensure that the spine specimens were located in the center of the cylinder, and then specimens were saved in −80°C and natural thawed 12 hours before the study.

#### 2.4.2. Instruments

Specially designed 5 mm two-tine SMA staples and implantation instruments as above were used.

#### 2.4.3. Experiment Grouping

The experiment groups were divided into four states: (1) a normal state (T6–T11 complete spinal specimens); (2) single staple state (T6–T11 spine specimens, a SMA staple was implanted across the right of the intervertebral space); (3) double staples state (two memory alloy orthopedic staples were implanted across the right of the intervertebral space); (4) postoperative single staple state (a 5 mm SMA staple activated as described in Section 2.3.4(2) was implanted across the right of the intervertebral space between every vertebral body (T6–T11) in normal living goat. Animals were sacrificed 4 months after the operation, and the fixed sections were removed. Six tests of spinal stiffness including the left and right lateral bending, left and right rotation, flextion. and extention were determined in each state as described above.

#### 2.4.4. Experiment Process

**Figure 7:**
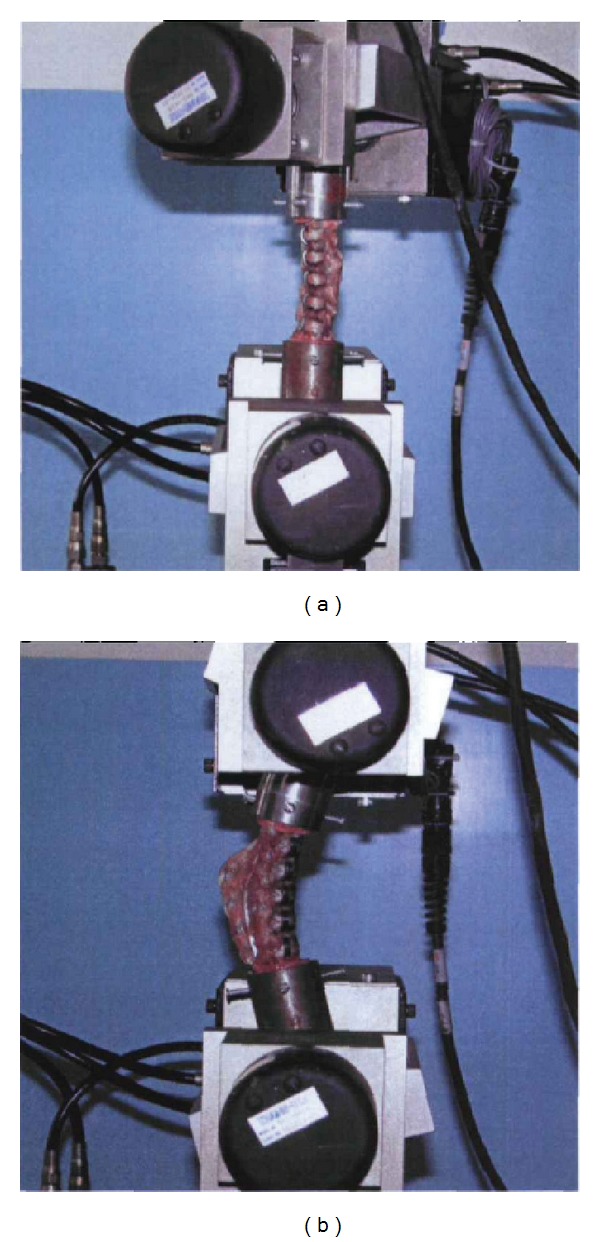
Test of the stability of the spine done physiological movement. (a) A right rotation; (b) flextion.

*(1) Test of Spinal Stiffness in Normal State (In Vitro).* The stability of the spine under normal state without staples was tested on the MTS858 Mini biomechanical test machine, with a spine fixtures 608.30,4-of DOF sensor. Experiment dedicated fixtures were fixed at the sensor's two terminals with screws, to ensure that the vertical center axis of the upper and lower fixtures was coincided in the same straight line, and that the horizontal axes were paralleled to each other. Spine specimens embedded were placed in the hollow cylinder of up and down ends of the fixture and fixed with screws. Before each test, the direction of the sensor had to be adjusted through the computer to ensure the correct position of the fixture. The torque and angle of goat thoracic specimens was controlled at the same time, so that the spine was applied to six physiological movement including the left and right rotation, left and right lateral bending, flextion, and extention (Figure 7). The movement would be stopped to make the specimen deformation recovered, once the sensor above or below the specimen reached any one of the control values of the torque and angle in the spinal movement process. The torque and angle control values of the sensor above and below, respectively, were left and right rotation ±1 Nm, ±30°, left and right lateral bending: ±6 Nm, ±15°, flextion: ±6 Nm ±20°, extention: ±3.5 Nm, ±20°. Data was sent out through the sensor, and then the overall movement of specimens was measured and recorded by analyzing system after the computer image processing. Each loading/unloading cycle was repeated three times, with the loading speed 1°/S, and in the third time kinematic measurements was carried out to reduce the influence of the viscoelasticity of the specimens. During the test processing, normal saline was used intermittently to keep the specimens moist. The spine stiffness values in six directions were derived from the raw data. If the stiffness values in any one direction increased, it indicated the segment stability. On the contrary, a decrease indicated the segmental instability.

*(2) Test of Spinal Stiffness in Single Staple State (In Vitro).* After the test in normal state was completed, the SMA staples were implanted in the right side of each adjacent vertebrae from 1 the vertebral transverse costal fovea anterolateral across the intervertebral space, with each intervertebral space one staple. Testing process and data calculate was the same with that in normal state.

*(3) Test of Spinal Stiffness in Double Staple State (In Vitro).* After the test in single staple state was completed, five SMA staples were implanted in the right side of each adjacent vertebrae from the anterolateral portion of staples implanted before across the intervertebral space that was two staples in each intervertebral space. Testing process and data calculate was the same with that in normal state.

**Figure 8:**
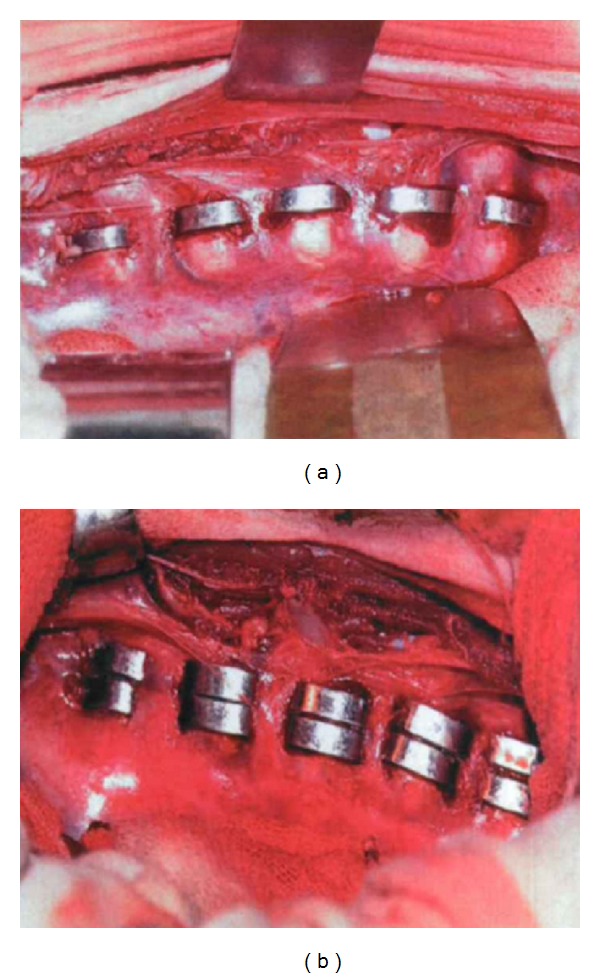
Intraoperative situation after the shape memory alloy orthopedic staples implanted. (a) Single staple group; (b) double staples group.

*(4) Test of Stability of the Spine in Postoperative Single Staple State (In Vivo).* Female goats of 2-3 months old, weighing 6–10 kg, were selected as specimens. Eight goats were treated with a same implantation surgery, and three goats without surgery were set as blank group. The surgical procedure was carried out as follows. The animals were anesthetized with intravenous injection of 3% pentobarbital sodium and then placed in left lateral position. The skin was straightly incised paralleled to the intercostal space between the sixth and seventh ribs and treated with electric coagulation. Then the subcutaneous tissue, the muscle tissue, and the rib periosteum under the surface layer of 7th rib were successively cut. Subperiosteal dissection was carried out to expose an 8 cm long section of rib, 1 which was cut and removed by a rib shear. After the periosteum and the parietal pleura below the rib were cut and the chest was open, the right lung showed an equal and satisfactory expansion. The thoracotomy device was installed to stretch the 6th and 8th ribs. The lungs under protection were retracted sidewards by dever retractors to reveal the thoracic vertebrae, which were arranged in neat rows, without side bend. A 5 mm shape memory alloy orthopedic staple was placed in ice water mixture and the two tines were stretched using distraction clamp to make the interdental distance 10 mm, which changed the original “C” shape of orthopedic nail into an open rectangular shape for use. The vertebral body and intervertebral space were identified. The bottom of the T6 vertebral body and the top of the T7 vertebral body in the anterior lateral portion of costotransverse joint were perforated using vertebral opening device across the T6 and T7 intervertebral space. Then the stretched shape memory alloy orthopedic staple held by distraction pliers was implanted into the channel of T6 and T7 vertebral bodies. Finally, the staple was beaten and compressed by a hammer device. Five 5 mm shape memory alloy orthopedic staples were implanted to the anterior portion of costotransverse joint and the anterior lateral of the vertebral bodies across the T6, 7, T7, 8, T8, 9, T9, 10, and T10, 11 intervertebral space successively (Figure 8(a)). In double-staple group, two 5 mm shape memory alloy orthopedic staples were implanted into right anterolateral of T6–T11 vertebral bodies across each intervertebral space successively, with a total of ten staples (Figure 8(b)). Covered by gauze treated with 55°C warm salt water repeatedly, the shape memory alloy orthopedic staples were deformed to restore the original shape. After rinsed, the 6th and 8th ribs were compressed closer using rib approximator. Before the chest was closed with double 7th suture, the tidal volume was increased to make the lungs swell and the residual gas was expelled. The muscles, subcutaneous tissues and skin were sutured successively and the incision was disinfected with iodophor. The surgery was completed. The intraoperative blood loss was about 30 mL to 50 mL during the surgery. 500 mL normal saline was injected intravenously and 3.2 million units of penicillin were applied. After the postoperative animals restored spontaneous breathing and chewing movements, the endotracheal tube was pulled out. Anterior X-ray and lateral X-ray was shot routinely. The postoperative animals were fed in sheepfold with outdoor activities every day. After 4 months, the goats were sacrificed and the fixed sections were removed for the spinal stiffness tests as above.

### 2.5. Statistical Analysis [21]

Statistical analysis was performed with SPSS software. Three sets of experimental results were assessed by random analysis of variance (one-way ANOVA) and pairwise comparison post hoc test. The significance level was set at *α* = 0.05.

## 3. Results

### 3.1. The Maximum Recovery Stresses of SMA Staples

**Figure 9:**
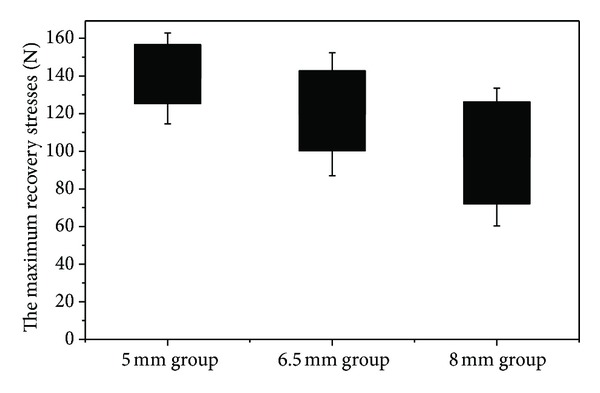
Comparison of the maxium recovery stresses among the three groups.

The maximum recovery stresses of SMA staples were determined as Figure 9. The maximum recovery stresses of SMA staples in 5 mm group, 6.5 mm group, 8 mm group were respectively 138.73 ± 12.05 N, 119.65 ± 16.34 N, 96.95 ± 18.27 N. Generally speaking, the statistical results in Table 1 showed there existed significant differences among these three groups (*P* < 0.01). Therefore, the 5 mm group exhibited the maximum of recovery stresses and the 8 mm group exhibited the minimum.

### 3.2. The Maximum Pullout Strength of SMA Staples

Comparisons of maximum pullout strength among the SMA staples and stainless steel groups were shown in Table 2. The maximum pullout strength in 5 mm group, 6.5 mm group, 8 mm group, and stainless steel group were, respectively, 74.18 ± 8.81 N, 51.28 ± 5.44 N, 39.13 ± 7.54 N, and 20.62 ± 9.15 N. There existed significant differences among these three groups by one-way ANOVA (*P* < 0.05). Therefore, the 5 mm group exhibited the maximum pullout strength and the 8 mm exhibited the minimum in the SMA staple groups. All the mean values of the three SMA staple groups were higher than that of stainless steel group.

### 3.3. The Results of Stability Test

The results of stiffness test were shown in Table 3. It indicated that in left and right bending, flextion, and extention, the stability of spine 1 was decreased in conditions of single staple and double staples compared to normal condition (*P* < 0.05). And in left and right rotation, there was no significant difference between those two conditions and normal one (*P* > 0.05).

### 3.4. The Postoperative Stability Test

**Figure 10:**
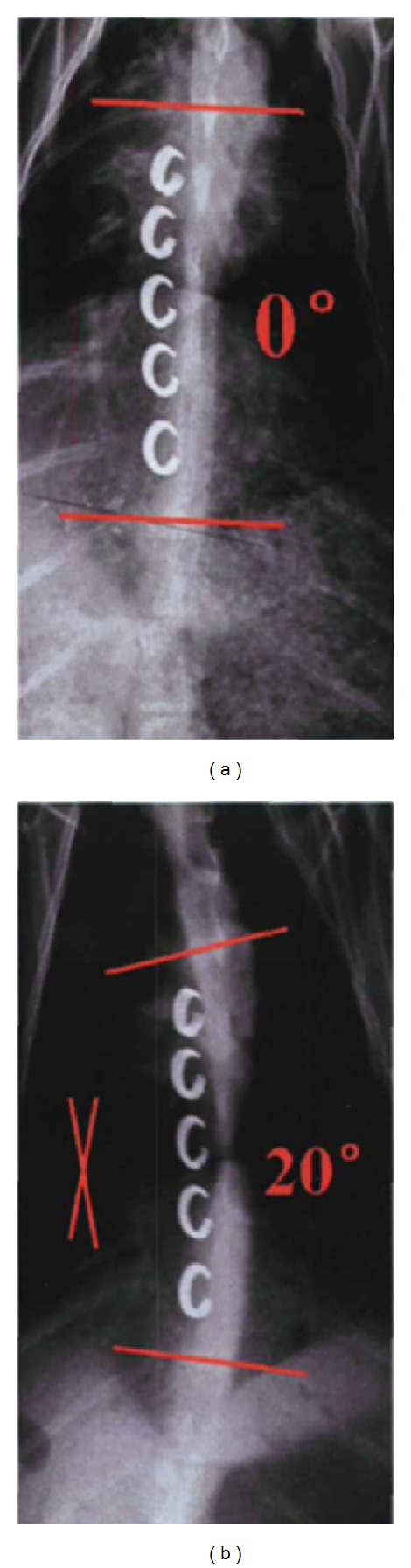
X-ray of the postoperative stability test. (a) After 2 months; (b) after 4 months.

The radiograph of one specimen was shown in Figure 10. It showed that after 4 months, a certain degree of scoliosis was developed *in vivo*. Through observation by X-ray, seven in eight of the goats developed scoliosis in different time, and two finally developed kyphosis. Therefore, the model-forming rates for scoliosis and for kyphosis were, respectively, 87.5% and 10.9%. Meanwhile, five in sixty SMA staples implanted into goat bodies were found loosing and shifting by continuous observation of X-ray. After the goats were sacrificed, the check of the staples showed that the shifting of staples was only developed to some extent, without dropping into the thoracic cavity from the vertebrae. The results of stiffness test of single staple state in Table 4 showed that 4 months after the SMA staples were implanted into the vertebrae bodies, the stiffness of the spine and the stability were increased. The stiffness of blank group (data not shown) was consistent with the normal state group above, which avoided the interference of growth.

## 4. Discussion

In recent years, goats, calves, dogs, pigs, and other large animals were used for research and development of the intrasegmental fixation instrument by more and more scholars [22–24]. In this research, goats at 2 to 3 months of age were selected. Two generations of memory alloy orthopedic staples in accord with the shape of the vertebral body of the goats were designed according to the anatomic features of the thoracic spine and the research about the implants depth and location of the staples. The metal surface on the first generation of staples did not made any treatment and the grooves in the tine of the second generation were designed to increase the resistance to pullout and rotation. For long-term stability and security of nickel-titanium alloy, surface coating and microporous design could be applied in the later clinical practices to prevent the release of nickel, increase bone ingrowth, and improve the long-term stability. 

The typical characteristic of memory alloy is shape memory effect, which is generally caused in martensite inverse phase change [25–27]. After heated to a temperature above the body temperature, the alloy generated inverse phase changed to restore its original shape immediately and produced great recovery stress to produce the orthopedic, pressure and bracing effects of the bone tissue [28]. Therefore, it was an important indicator for evaluation of shape memory effect. In this research, three groups (5 mm, 6.5 mm, and 8 mm) of two-tine memory alloy orthopedic staples were tested and the results showed that the 5 mm staples exhibited the greatest average values of maximum recovery stress and the 8 mm orthopedic staples exhibited the least. There were significant differences in the average values among the three groups. Therefore, the maximum recovery stress of memory alloy orthopedic staples exhibited an anticorrelation with the original distance between the tines. It meant that when the distance between the tines became smaller, the relative displacement of the original tines were larger which caused greater values of recovery stress. It possibly attributed to the transformation of the crystal sequence structure and the cooperation-displacement shear of the atoms on the interface.

The early stability of any intrasegmental fixation instrument implanted into bodies is crucial to the success of the surgery and it is an important evaluation method to test the pullout strength [29, 30]. The factors affecting the pullout strength included the direction of load, the method of implanting intrasegmental fixation, the length of intrasegmental fixation, and bone quality. In clinical practice, the direction of load pullout was not always consistent with the axis of intrasegmental fixation. If the intrasegmental fixation was pulled out at some angle, the bone could be ruined and the direction was not easy to control. Therefore, vertical pullout was used in this research to make sure the axis of load consistent with the implanting direction of SMA staples. In order to avoid the influence of the bone, the spine of goats at the age of 2 months were selected for specimens. In this research, unified staple implanting method by which the orthopedic staples were hammered into the functional sections of spine was used. The results showed that the stainless steel exhibited the minimum average values of the maximum pullout strength. The 1 average values of the maximum pullout strength were increased with the decrease of the interdental distance, which were related to the recovery stress. Therefore, shape memory effect was the basis of pullout strength of the memory alloy orthopedic staples. In this research the pullout strength of stainless steel staple was compared with that of the three SMA staples under the same condition, which showed that the antipullout performance could be increased by changing the material of intrasegmental fixation.

Spinal stability refers to the ability of spine to maintain its own balance position, which reflects the relationship between the load and the displacement caused by the load. Under the same load, the smaller the displacement changes, the stronger the stability becomes. From the view of biomechanics analysis, spinal instability refers to the spinal activity abnormality increases, or the decrease of the FSU stiffness. Stiffness is the spinal ability of resistance to deformation, which is one of the indicators for evaluation of the carrying capacity of spine. The stiffness test of spinal fixed segment is commonly used for biomechanical stability test of intrasegmental fixation instrument *in vitro* [31, 32]. This experiment was carried out *in vitro* environment. Memory alloy orthopedic staples were implanted into right sides of T6–T11 thoracic vertebrae and the stability test of six physiological movements including the left and right rotation, left and right lateral bending, flextion, and extention was achieved. Results showed that, along the axis of rotation, whether to implant the memory alloy orthopedic staple or how many staples to be fixated would not increase or decrease the stability of the spine, whereas on the directions of lateral bending (left/right), flexion, and extension, the stability of single staple state and double staple state of the spine samples was lower than that of the normal state. This might be explained that due to the implantation of the memory alloy orthopedic staples and its corresponding recovery stress, the cancellous bones at one side of the vertebrae were compressed; an effect approximates to a slight wedge-shaped deformity on the side of the vertebrae. Upon right lateral bending and flexion of the spine samples, the rift between the outer surface of the memory alloy orthopedic staples and the cancellous bones of the vertebrae became narrower; thus the staples played a role here as a leverage, increasing the range of motion of the left and rear intervertebral disc, as a result the entire stability of the spine was decreased. Upon left lateral bending and extension, the deformity in the right and frontal intervertebral disc was in consistence with that of the normal state, whereas the rift between the outer surface of the memory alloy orthopedic staples and the cancellous bones of the vertebrae was enlarged, causing the separation movement of the staples and bones. This might also increase the range of motion of the entire spine and decrease its stiffness. Betz et al. [33] have carried out similar experiments on the thoracic vertebrae of the bovine and to a certain degree verified that *in vitro* anterior implants of fixtures did not necessarily increase the stability of the spine. 

Although the immediate stiffness test *in vitro* showed a decrease of spinal stability, the postoperative stiffness test after 4 months presented a restoration of the stability. The cause of the recovery may be as follows. (1) In the living state, the bones of vertebral body could be repaired and rebuilt by themselves, which could narrow the interspace and alleviate the movements between the metal surface and the vertebral body. (2) The appearance of the connective tissue and new bones around SMA staples remedied the adverse effects caused by lateral compression of bones. (3) The structure of ligament could be repaired and the balance could be rebuilt.

## 5. Conclusions

In this research, two kinds of SMA staples were successfully designed. SMA staples presented better antipullout performance, which was superior to stainless steel staple. *In vitro*, there existed an instant decrease of stability with the implantation of SMA staples. However, the spine could increase its stiffness and stability after the SMA staples were implanted *in vivo* for certain times and partial functions were also reserved in the fixed segments. SMA staples which have the function of hemiepiphyseal compression could be also used for kyphosis and scoliosis model of thoracic vertebrae in goat.

## Figures and Tables

**Table 1:** One-way ANOVA of the maximum recovery stresses.

	Sum of squares	DF	Mean square	*F* value	*P* value
Intergroup comparison	6999.722	2	3499.861	14.077	0.000
Intragroup comparison	5221.128	21	248.625	/	/
Overall	12220.850	23	/	/	/

**Table 2:** Comparison of the maximum pullout strength.

Staples	*N*	Maximum pullout strength	Minimum	Maximum
Stainless	24	20.62 ± 9.15	9.49	37.78
8 mm	8	39.13 ± 7.54	27.85	50.93
6.5 mm	8	51.28 ± 5.44	44.16	60.52
5 mm	8	74.18 ± 8.81	59.63	87.84

**Table 3:** Results of stiffness tests and statistical comparison among three states.

Movement	State	Mean values of stiffness (Nm/deg)	*P* value*
Right rotation	Normal	0.035 ± 0.013	>0.05
	Single staple	0.022 ± 0.005	
	Double staple	0.028 ± 0.009	

Left rotation	Normal	0.038 ± 0.009	>0.05
	Single staple	0.030 ± 0.008	
	Double staple	0.028 ± 0.009	

Right bending	Normal	2.38 ± 0.22	(N&S) <0.05
	Single staple	1.8 ± 0.12	(N&D) <0.05
	Double staple	1.69 ± 0.13	(S&D) >0.05

Left bending	Normal	2.57 ± 0.15	(N&S) <0.05
	Single staple	1.42 ± 0.27	(N&D) <0.05
	Double staple	1.19 ± 0.12	(S&D) >0.05

Flextion	Normal	2.42 ± 1.14	(N&S) <0.05
	Single staple	1.04 ± 0.35	(N&D) <0.05
	Double staple	0.99 ± 1.21	(S&D) >0.05

Extention	Normal	2.57 ± 0.15	(N&S) <0.05
	Single staple	1.42 ± 0.27	(N&D) <0.05
	Double staple	1.19 ± 0.12	(S&D) >0.05

*S stands for single staple state, D stands for double staple state, and N stands for normal state.

**Table 4:** Stiffness tests of postoperative single staple state and statistical comparison of the postoperative single state staple with single staple, double staple, and normal states.

Movement	Mean values of stiffness (Nm/deg)	*P* value*
Right rotation	/	(S2&N) >0.05 (S2&D) >0.05 (S2&S) >0.05

Left rotation	/	(S2&N) >0.05 (S2&D) >0.05 (S2&S) >0.05

Right bending	3.27 ± 0.37	(S2&N) <0.05 (S2&D) <0.05 (S2&S) <0.05

Left bending	2.51 ± 0.43	(S2&N) >0.05 (S2&D) <0.05 (S2&S) <0.05

Flextion	2.58 ± 0.24	(S2&N) >0.05 (S2&D) <0.05 (S2&S) <0.05

Extention	1.87 ± 0.07	(S2&N) >0.05 (S2&D) <0.05 (S2&S) <0.05

*S2 stands for postoperative staple state, S stands for single staple state, D stands for double staple state, and N stands for normal state.
